# Intranasal delivery of a polymeric nanoparticle subunit vaccine for the induction of protective immunity against respiratory syncytial virus

**DOI:** 10.3389/fimmu.2026.1824823

**Published:** 2026-06-26

**Authors:** Sarah M. Ostrowski, Paul R. Hartmeier, Madeline A. Lipp, Timothy Perkins, Elliot Reed, Xiyue Li, Wilson S. Meng, Kerry M. Empey

**Affiliations:** 1 Department of Pharmacy and Therapeutics, School of Pharmacy, University of Pittsburgh, Pittsburgh, PA, United States; 2 Center for Clinical Pharmaceutical Sciences, School of Pharmacy, University of Pittsburgh, Pittsburgh, PA, United States; 3 Graduate School of Pharmaceutical Sciences, School of Pharmacy, Duquesne University, Pittsburgh, PA, United States; 4 Department of Pathology, School of Medicine, University of Pittsburgh, Pittsburgh, PA, United States; 5 McGowan Institute for Regenerative Medicine, University of Pittsburgh, Pittsburgh, PA, United States; 6 Department of Immunology, School of Medicine, University of Pittsburgh, Pittsburgh, PA, United States; 7 Center for Vaccine Research, School of Medicine, University of Pittsburgh, Pittsburgh, PA, United States; 8 Clinical and Translational Science Institute, School of Medicine, University of Pittsburgh, Pittsburgh, PA, United States

**Keywords:** dendritic cells, intranasal, mucosal immunization, nanoparticle vaccine, prefusion F protein, resident memory T cells, respiratory syncytial virus

## Abstract

Respiratory syncytial virus (RSV) is the leading cause of lower respiratory tract infection in children under the age of 5, worldwide. Maternal vaccination and monoclonal antibodies provide only temporary protection for young children. As antibody wanes, young children remain at risk for severe RSV disease, creating an unmet need for a direct childhood vaccine. We developed an intranasal (IN) subunit vaccine, comprised of a biotinylated nanoparticle and RSV prefusion protein DS-Cav1 (PreF-bNP) with a flexible “plug-n-play” design that enables dose optimization of antigen and adjuvants. In a preimmune RSV mouse model, immunization with intranasal PreF-bNP increased IL-12+ dendritic cells, generated Th1- polarized CD4 effector and tissue resident memory T cells (TRMs) and established antiviral CD8 TRMs in the lung. PreF-bNP-mediated antiviral responses correlated with complete RSV protection up to 8 weeks with reduced mucus production in the lungs. Together, the data presented demonstrate that intranasal PreF-bNP safely protects against RSV infection in young preimmune mice and should be further investigated for future clinical development.

## 1 Introduction

Respiratory syncytial virus (RSV) infects nearly 100% of children by age two. Each year, RSV causes ~33 million episodes of acute lower respiratory illness and is responsible for over 101,000 deaths in children up to five years of age (1, 2). In a single year, 3.6% of all deaths in children under 6 months of age were attributed to RSV (3). While prevention of severe RSV disease in this age group is now possible with the approved maternal vaccine (Abrysvo, Pfizer) –and long-acting monoclonal antibodies, nirsevimab and clesrovimab – the duration of protection is limited to ~5–6 months (4–6) or a single RSV season in a child’s first year of life (7). Consequently, children beyond this window of protection remain vulnerable to primary and repeated RSV infections, thus there remains an urgent and unmet need for a vaccine that provides safe, long-term RSV protection in young children.

While live attenuated vaccines can effectively generate protective immunity, they are also linked to the rare, but real risk of causing life-threatening infections (8). Particularly important for young children, subunit vaccines avoid this safety risk and bring the added advantages of focused oligo-clonal immune responses and enhanced process controls in large scale manufacturing (9). The lack of inherent danger signals in recombinant protein antigens necessitates the co-formulation of subunit vaccines with immune stimulating adjuvants. A current paradigm is that maximal immunity occurs when the adjuvant and subunit antigens are both associated with the same particle (10), colocalized to the same antigen presenting cell (11), and drain to the same lymph nodes (12). Spatiotemporal colocalization of antigen and adjuvant components is reported to activate both dendritic cells (DCs) and B cells (13), resulting in antigen presentation and stimulation of antiviral CD4 helper T and CD8 cytotoxic T cells. Coordination of antigen presentation signals promotes induction of tissue resident memory T cells (TRMs) (14), which are primed for rapid reactivation at the site of infection. These observations have led to formulation strategies in which the antigen and adjuvant are physically or chemically coupled (15, 16). Coupling of the two principal vaccine components can improve patient safety by narrowing the immune response towards the target antigen and limiting inflammation to immediate locoregional tissues (17).

We previously described the vaccine delivery attributes of a biotinylated nanoparticle system (referred to as bNP) formed by co-emulsification of poly (d,l-lactic-co-glycolic acid) (PLGA) and the lipid 1,2-distearoyl-sn-glycero-3-phosphoethanolamine (DSPE) conjugated with PEG(2000)-biotin. PLGA polyesters are biodegradable (18, 19) and for decades have been used in medical applications including in sutures and in long-acting injectable pharmaceutical formulations (19, 20). PLGA nanoparticles have been investigated extensively in preclinical settings as vehicles for subunit vaccines, due in part to their facile manufacturing process, preferential uptake by antigen presenting cells (APCs), and potential for facilitating cross-presentation of antigens by DCs for the activation of CD8 T cells (21–23). bNP is decorated with surface biotin, thereby enabling loading of a variety of antigens and adjuvants and personalized dose optimization using streptavidin (SA) or avidin as the linker. Surface loading spares protein antigens from denaturation that can occur during the more traditional encapsulation process (24). DS-Cav1 (referred to from here on as PreF), the RSV fusion protein stabilized in its highly antigenic prefusion conformation, was biotinylated and conjugated to the novel bNP platform to create an RSV subunit vaccine (PreF-bNP).

Nanoparticles can efficiently traverse mucosal barriers and deliver antigens directly to the nasal epithelium, positioning intranasal vaccination as a powerful strategy against respiratory pathogens that initiate infection in the upper airway. Preclinical studies have shown that intranasal immunization provides a stronger mucosal immune response against respiratory viruses than conventional intramuscular delivery by generating rapid, site-specific immunity capable of preventing infection in the upper and lower respiratory tracts (25–27). Here we describe the immune response and viral protection generated from intranasal PreF-bNP vaccination in a preimmune mouse model that reflects human RSV exposure in young children.

## 2 Materials and methods

### 2.1 Nanoparticle preparation and characterization

bNP were prepared as described previously (15). Briefly, PLGA 50:50 (PolySciences Inc., Warrington PA) and1,2-distearoyl-sn-glycero-3-phosphoethanolamine-N-[biotinyl(polyethyleneglycol)-2000] (DPSE-PEG(2000)-biotin (Avanti Polar Lipids, Alabaster, AL)) were dissolved in methylene chloride to create the organic phase, then added dropwise into 1% w/v PVA during homogenization, the final oil-in-water emulsion was allowed 4 hours for methylene chloride evaporation prior to lyophilization. bNPs used for Near IR imaging were tagged with 5mM of CellVUE NIR815 by including it during the organic phase. Particle size distribution and zeta potential were measured using a Malvern ZS Nano (Malvern, Malvern, UK) at 0.3 mg/mL in de-ionized H_2_O. Stability was determined by suspending bNPs at 10 mg/mL in PBS (pH 7.2), storing them at 4 °C and measuring size once a week for up to 4 weeks. Scanning electron microscopy (SEM) images were captured on a Zeiss Sigma VP SEM (Oberkochen, Germany) at 1kV accelerating voltage and 50,000x magnification.

### 2.2 DS-Cav1(PreF) Biotinylation and validation

DS-Cav1, hereon referred to as PreF (Calder Biosciences, Brooklyn, NY), was biotinylated using EZ-link NHS-PEG4-biotin (ThermoFisher, Waltham, MA) at 10mmol NHS to 1mmol PreF. The reaction proceeded at 4 °C for one hour. The samples were dialyzed in a Slide-a-Lyzer Tube (ThermoFisher, Waltham, MA) at 4 °C for two hours with PBS followed by buffer exchange and dialysis for an additional two hours to yield biotinylated PreF. 4’-hydroxyazobenzene-2-carboxylic acid (HABA) displacement assay was used to confirm biotinylation and the molar ratio of biotin:PreF. 300 µM HABA per0.5 mg/mL avidin was prepared and absorbance measured at 500 nm before and after the addition of biotinylated PreF. The molar ratio of biotin:PreF was determined using the HABA/avidin extinction coefficient of 34,000 M^-1^*cm^-1^ at 500 nm. Conformation and binding of biotinylated PreF was determined using the human anti-RSV PreF antibody AM14 (Cambridge Biologics, Brookline, MA) and PE-goat anti-human Fc antibody (Invitrogen, Waltham, MA). PreF-bNP was centrifuged, and the supernatant analyzed for unbound AM14 at 565/576 nm (ex/em) on a Tecan microplate fluorescent reader.

### 2.3 PreF-bNP size distribution, zeta potential, and AM14 capture assay

bNP was loaded with biotinylated PreF via a SA linker and evaluated at 4 °C for up to four weeks. At weekly timepoints, the complexes were evaluated for particle size distribution, zeta potential, and PreF surface loading and stability by AM14 capture as described above. At t=0, PreF-bNP SEM images were captured on a Zeiss Sigma VP SEM (Oberkochen, Germany) with at 1kV accelerating voltage at 5,000 and 10,000x magnification. Samples were prepared by rinsing the bNPs of trehalose using the methods described above and then loaded with biotinylated PreF as described above. The samples were then washed with DI H_2_O and dried on a silicon wafer prior to imaging.

### 2.4 Imaging and image analysis

C57BL/6 or BALB/c mice (Hilltop Lab Animals, Scottsdale, PA) were imaged using an Odyssey Pearl Imager at 800 nm, with a resolution of 170 µm for all images after IN administration. All mice were kept in a pathogen-free facility, and all animal use protocols were approved by Duquesne University Institutional Animal Care and Use Committee.NIR815 and 680RD were used for detection of nanoparticles and SA respectively. The mean fluorescent intensity (MFI) of each channel in the mouse lungs and lymph nodes was determined using Li-Cor Image Studio V5.5 with the small animal module. For images of mice where only the chest plate was removed to expose the organs, background subtraction was performed using the left footpad as control and areas of fluorescence were manually defined by using the “Draw Freehand” tool to quantify fluorescent intensity above background. For images where organs were imaged ex vivo, the imaging stage was used for background subtraction and the “Draw Freehand” was used to define area of fluorescence.

### 2.5 Flow cytometry

BAL cells were incubated for 3 hours at 37 °C in 10% RPMI with 1:1000 Brefeldin A. After incubation cells were washed with HBSS and then were surface stained to identify innate cells with a combination of the following antibodies (clone): LIVE/DEAD Fixable Blue, CD16/32 (2.4G2), SiglecF (E50-21440), F4/80 (T45-2342), CD11b (M1/70), Ly6G (1A8), CD11c (N418), MHCII (M5/114.15.2), CD45 (30-F11). Cells were fixed and intracellularly stained with IL-12 (C15-6) and IL-10 (JES5-16E3) prior to analysis. Circulating and resident memory T cells from BAL and whole lungs, respectively, were stained with the following antibodies: CD44 (IM7), CD11a (M17/4), CD49a (HMa1), CCR7 (4B12) CD103 (2E7). Cells were fixed and intracellularly stained with: IL-13 (eBIO13A), Granzyme B (QA16AO2), IL-5 (TRFK5), and IFNγ (XMG1.2) and run on a Cytek Aurora managed by the United Flow Core of the University of Pittsburgh. Data was analyzed using FlowJo V10 software (FLOWJO, LLC, Ashland, OR). The gating strategies for innate and T cell data are shown in **Supplementary Figure 3** and **Supplementary Figure 4**, respectively.

### 2.6 Imaging flow cytometry

Six-week-old Balb/C mice were IN infected with 4x10^5^ pfu/g RSV. 1 week post infection, mice were intranasally administered PreF-bNP or biotinylated PreF conjugated to PE-Dazzle594-tagged SA. 2 days post vaccination, BAL was collected from mice and cells were surface stained with a combination of the following antibodies: CD16/32, MHCII, CD11c, and F4/80 (same clones as flow cytometry). On the day of analysis, cells were then stained with DRAQ5 to assess viability. All samples were run on the ImageStream^X^ MKII managed by the Unified Flow Core at the University of Pittsburgh, and data was analyzed using IDEAS software v6.3 (Luminex Corporation, Seattle, WA). The compensation matrix was adjusted based on single color compensation beads and applied to all samples. Area versus aspect ratio was used to gate out debris and identify single cells. The cell boundaries were defined using the Adaptive Erode Mask tool from the BrightField (BF) image – the established Internal Mask was then applied to all gated cells. The internalization probe was set to PE-Dazzle 594 (Channel 4) to indicate tagged biotinylated PreF or PreF-bNP. The percent internalization of antigen or particles was calculated by Equation 1.

(1) $$\%\;Internal=(\frac{Fluorescent\;Intensity\;in\;Internal\;Mask}{Fluorescent\;Intensity\;})*100$$

Where Intensity in Internal Mask refers to PE-Dazzle594 fluorescence greater than 50% detected within the set Adaptive Erode Mask, and Fluorescent Intensity is the fluorescent intensity of PE-Dazzle594 within the entire detection channel.

### 2.7 Mice, RSV infection, and intranasal immunizations

BALB/c mice (The Jackson Laboratory, Bar Harbor, ME) were bred 1:1 for the timed delivery of pups. All mice were maintained in a pathogen-free facility at the University of Pittsburgh, Division of Laboratory Animal Resources (Pittsburgh, PA) and handled according to protocols approved by The University of Pittsburgh Institutional Animal Care and Use Committee. On postnatal day (PND) 7 pups were infected intranasally (IN) with 2.5x10^5^ pfu/g of RSV L19. Three weeks after infection, pups were treated with PBS, 10μg PreF, or 10μg biotinylated PreF/1mg lyophilized bNP powder (PreF-bNP) IN; each formulation was resuspended in or q.s to 100 μL of PBS. Three weeks later, each group was boosted with their respective vaccine doses or PBS control. In some mice, lungs were harvested 4-weeks after the boosting dose for analysis of resident memory T cells (TRMs). At 4- or 8-week post-boost the remaining mice were challenged with 5x10^5^ pfu/g of RSV L19.

### 2.8 Viral titers

Left lungs were collected from mice at each timepoint for viral titers as previously reported (28). Briefly, the left lungs were harvested, flash frozen, and stored at -80 °C prior to analysis. After thawing at 37 °C, lungs were ground with glass by mortar and pestle, centrifuged 2000 RPM for 15 minutes, and 100μL of supernatant was incubated on a HEP-2 monolayer under 0.75% methylcellulose in Minimum Essential Medium (MEM) with 10% FBS for 5 days. After incubation, the cells were fixed and stained with hematoxylin-eosin for plaque quantification.

### 2.9 Modeling and simulations

The elimination rates of SA and bNP were quantified in lungs and draining lymph nodes (dLN) after intranasal administration by fitting *in vivo* imaging data to a first-order equation (MATLAB 2018, Natick, MA). *In vivo* near-infrared signal intensities over 7 days were quantified and converted to concentrations using the intensity obtained at time of administration of SA and biotin in bNP. A two-compartment model was built assuming elimination from the lungs into the dLN or into an undefined central compartment. The dissociation of SA from biotin was included in the model (29). Data was fitted to the model using *fminsearch* with coefficient and function termination tolerances of 1x10–^8^ and 400 max iterations. Evaluation of the model was performed by running simulations with the final fitted parameters by inputting specific doses, either from the observed *in vivo* data or varying for the purpose of dose ranging.

### 2.10 Data analysis

Zeta potential, size, and Near IR imaging, were performed with technical replicates N = 3–5 and analyzed by one-way ANOVA with Tukey’s *post hoc* test for comparison of multiple groups. Flow cytometry was performed with biological replicates of N = 6–8 per group and was analyzed via two-way ANOVA with a full model fit and Tukey’s *post hoc* analysis for multiple groups. ImageStream was performed with N = 4 biological replicates for each group with BAL of 2 mice being combined to create one sample (total 2 samples per group). All data are presented as mean± standard error of the mean (SEM). Statistical analysis for all experiments was performed using GraphPad Prism 10 (San Diego, CA) with a significance threshold of *p* < 0.05.

## 3 Results

### 3.1 Stability of biotinylated prefusion antigen on bNP

**Figure 1:**
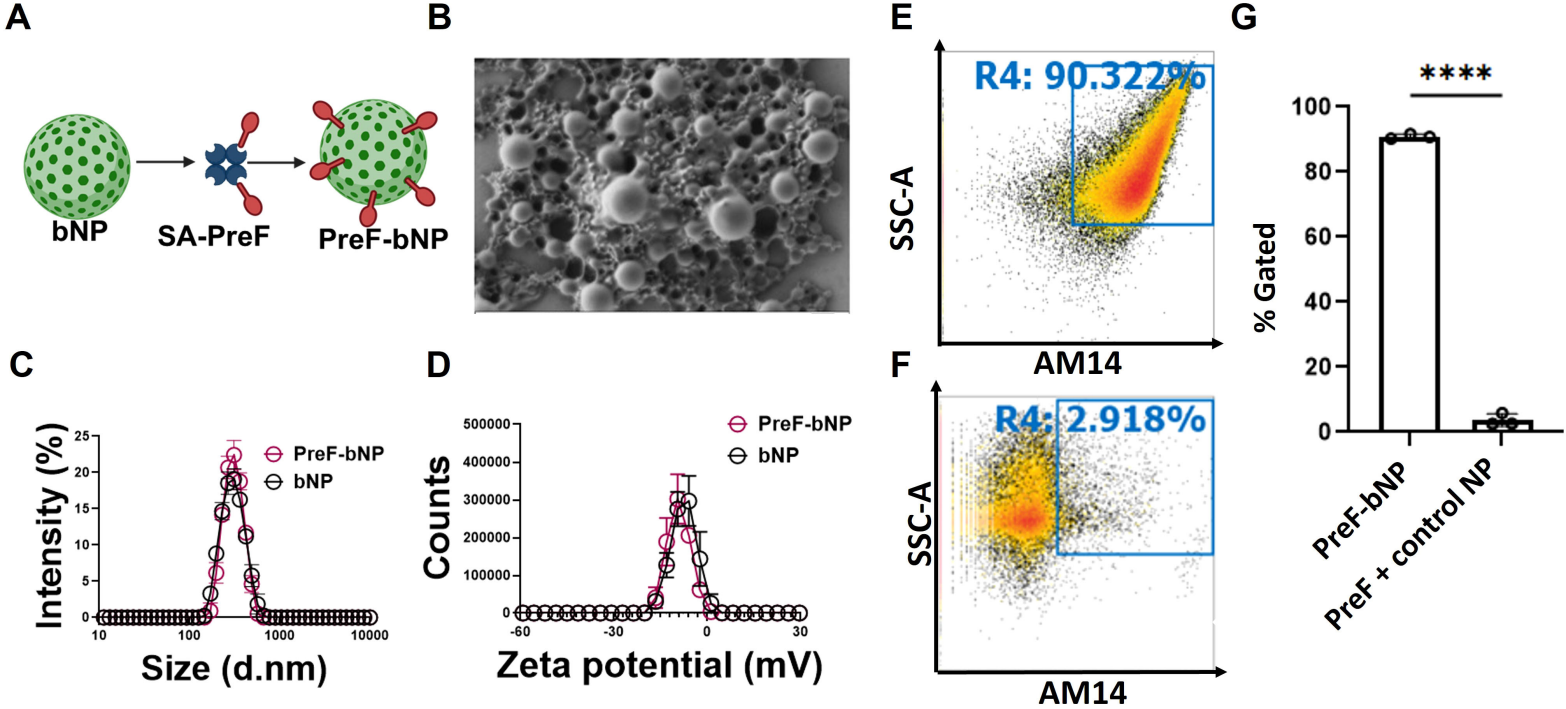
PreF-bNP is colloidally and antigenically stable for up to 1 week at 4 °C **(A)** Representative diagram of PreF-bNP formulation - created by using BioRender. **(B)** SEM micrographs of PreF-bNP at 15,000x magnification. **(C)** Size and **(D)** zeta potential of PreF-bNP versus bNP alone. **(E)** Representative flow cytometry gating of percent PreF+ bNP **(F)** Representative flow plot of percent PreF+ control PLGA nanoparticle. **(G)** Percent PreF pulled down from PreF-bNP or control nanoparticles from flow cytometry. ****p<0.0001 by unpaired t-test.

Loading of the RSV antigen PreF on bNP was accomplished by heterologous biotin-SA-biotin interactions in which the four binding sites of SA are occupied by biotinylated PreF and biotin molecules on bNP (PreF-bNP) (**Figure 1A**). Stability of the particles after antigen loading was confirmed using dynamic light scattering for particle size and surface potential measurements. The particles remained intact and spherical in the dehydrated state under SEM (**Figure 1B**). The mean size of bNP at t=0 was 420 nm with zeta potential of -3.40 mV and PreF-bNP was 550 nm (**Figure 1C**) with a zeta potential of -7.96 mV (**Figure 1D**). PreF-bNP particle size remained stable in phosphate buffer for up to one week at 4 °C but significantly increased by 4 weeks. zeta potential ranged from -5 to -10 mV (**Supplementary Figures 1a, b**). These results indicate that antigen-loaded bNP are stable colloids.

The specific conformation of biotinylated PreF on the bNP particle surface was evaluated over the same four-week period. Bound biotinylated PreF was detected using the AM14 antibody for protein pulldown assay. The percent capture of biotinylated PreF by the antibody at t=0 was 88%, with a nonsignificant reduction in antibody capture through 4 weeks (**Supplementary Figure 1c**). To confirm that biotinylated PreF remained tethered to bNP, flow cytometry was used to compare PreF-bNP against control, non-biotinylated PLGA nanoparticles incubated with PreF and SA after 1 week. Utilizing an anti-Fc secondary antibody conjugated to a fluorescent dye, the presence of a conformationally stable biotinylated PreF on the particle surface was confirmed with ~90% of particles exhibiting the fluorescent tag (**Figures 1E, F**). Conversely, biotinylated PreF and SA incubated with non-biotinylated PLGA NPs exhibited less than 10% of particles exhibiting fluorescence (**Figure 1G**). Taken together, the PreF-bNP formulation is colloidally and antigenically stable for up to one week at 4 °C, in line with many reconstituted licensed vaccine products (30, 31).

### 3.2 Spacing of PreF on bNP surface

**Figure 2:**
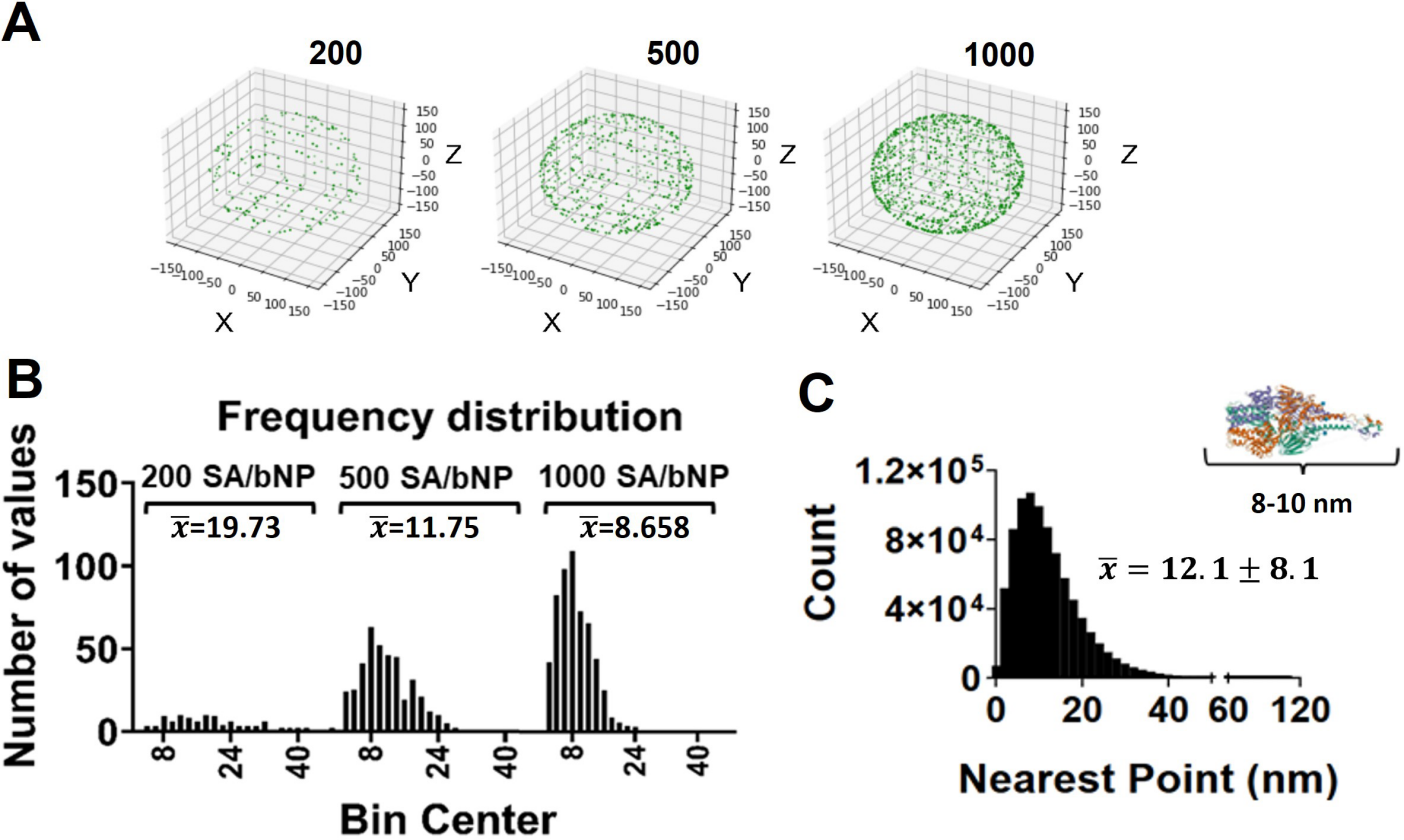
Theoretical basis of surface loading of PreF on bNP. **(A)** Simulated bNP surface plots with 200, 500, or 1000 SA molecules on surface. **(B)** Nearest Neighbor histograms of points on bNP surface from left to right, 200 SA/bNP with an average distance of 19.73 nm, 500 SA/bNP with an average distance of 11.75 nm, and 1000SA/bNP with an average distance of 8.658 nm. **(C)** Nearest neighbor histogram of entire bNP population and size of PreF protein. Engineered RSV Prefusion antigen DS-Cav 1 PDB code: 4MMU. The computation was performed using a set of custom Python codes.

A computational analysis was performed to estimate the spacing of antigen loading on bNP surface. Random point distributions were determined computationally based on experimental measurements of bNP particle size, where each bNP particle should have 8.0 pmol biotin/mg of solvent accessible biotin molecules (15). Simulations of biotin binding sites were performed based on theoretical loading 200, 500, and 1000 molecules of SA per particle (**Figure 2A**). The nearest neighbor between each point on the surface, assigned as arc length, was an average distance of 19.73 nm, 11.75 nm and 8.87 nm for the 200, 500 and 1000 SA molecules loaded, respectively (**Figure 2B**). The number of biotin molecules on the particle surface, or particle surface density (PSD), was estimated from the moles of SA pulled down experimentally, the known mean diameter of the bNP, and the estimated density of the materials (**Supplementary Figures 2a–d**). Dispersity index values (PDI) (<0.2) were incorporated into the simulation to render a population-based approach. The average distance between each biotin on the surface of bNP was calculated to be 12.1 nm± 8.1. Given the estimated lateral dimension of PreF trimer approximated to be 8–10 nm (32), this allows surface loading without steric overlap (**Figure 2C**). The computational outcome is consistent with the observed colloidal stability; the simulation also provides a theoretical upper limit of antigen loading on the surface.

### 3.3 Retention of antigen in lung and draining lymph nodes enhanced by bNP

**Figure 3:**
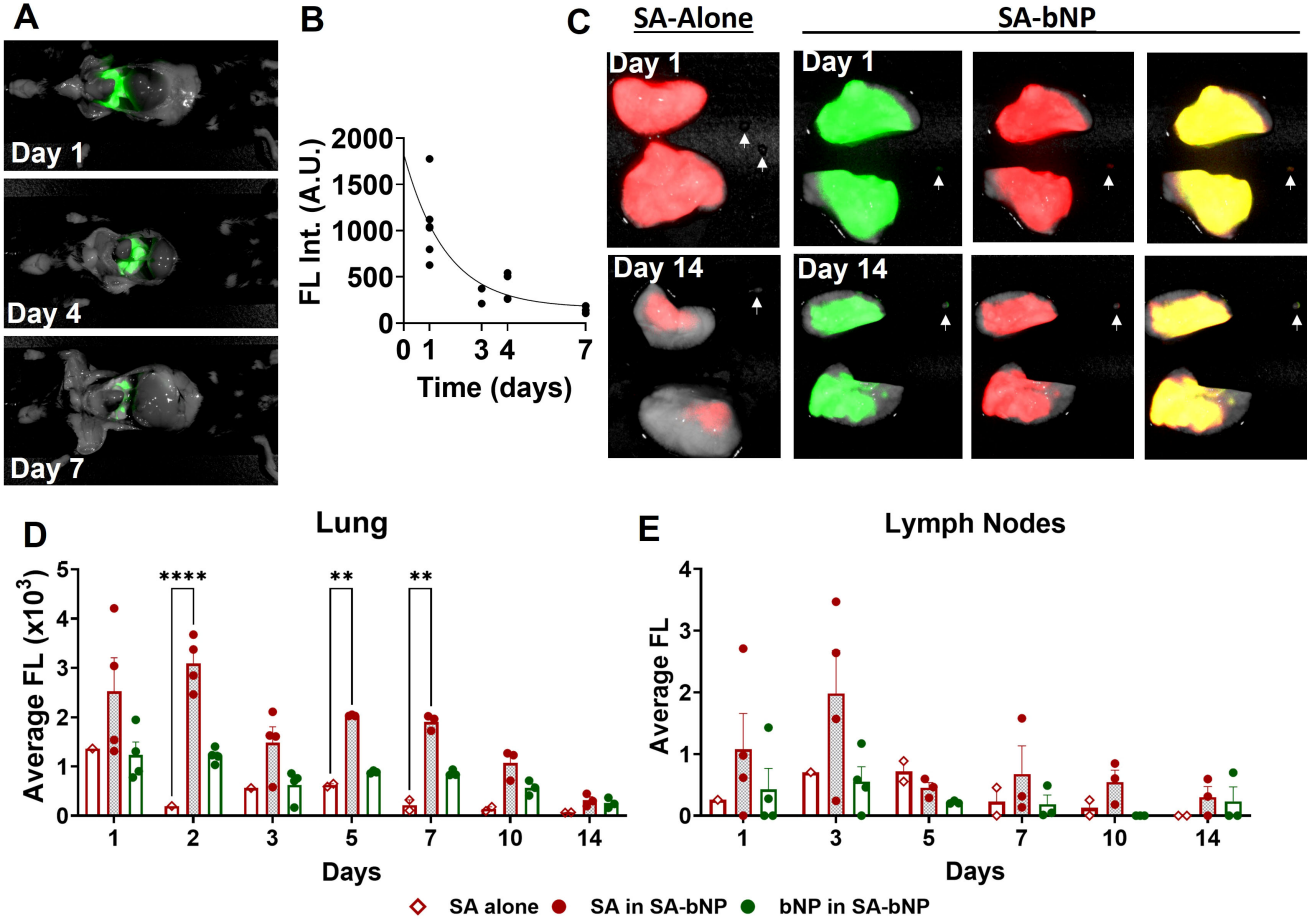
bNP enhances antigen retention in the lungs. **(A)** Representative images of mice with fluorescent lungs after being IN-administered 1 mg of NIR bNPs at 1-, 4- and 7-day post-administration. **(B)** Fluorescent intensity in the lungs vs time up to 7 days. **(C)** Representative images of fluorescent lungs and dLN (white arrows) of SA alone and SA-bNP at 1- and 14-days post IN administration. **(D)** Average fluorescence of right and left lungs per mouse. **(E)** Average fluorescence of all dLN collected from each mouse (1-2dLN/mouse). N = 1–2 mice SA alone per timepoint, N = 4 SA-bNP per timepoint. **p<0.01; ****p<0.0001 by 2-Way ANOVA followed by a Tukey’s multiple comparison.

The retention kinetics of bNP *in vivo* were determined using fluorescent dye labeled PreF-bNP in mice. bNP was encapsulated with NIR815 dye via the organic phase of manufacturing (NIR815-bNP). 1mg of NIR815-bNPs were administered intranasally in 100μl of PBS to adult C57BL/6 mice. Lungs were collected from inoculated mice at 1-, 3-, 4- and 7-days and imaged using Li-Cor Pearl Imager. High levels of fluorescence were observed on day 1, which decreased through day 3 and day 7 (**Figure 3A**). The distribution of fluorescence appears to be relatively equal between both the left and right lungs, with limited signal observed in the stomach at each time point. The intensity plots were fitted to a first-order elimination function, and the t=0 concentration was estimated from that curve. These results support the notion that bNP is efficiently delivered into the lungs via the IN route. Elimination of bNP from the lungs followed pseudo first-order elimination kinetics when the fluorescence is fitted vs time (**Figure 3B**) from which an elimination rate constant of 0.78 days^-1^ was calculated, and a resultant half-life of 0.9 days was estimated.

BALB/c mice were intranasally treated with 1mg of NIR815-bNP (“green”) complexed to 2μg of SA conjugated with 680RD (“red”) dye suspended in 100μL PBS. Distribution was compared to mice intranasally treated with equivalent amounts of SA via imaging on the Li-Cor Pearl Imager. Previously, we saw SA-specific B cells were induced after subcutaneous administration of SA-bNP, therefore SA would be used as a mock antigen to examine delivery and retention (16). Mice were intranasally administered SA alone or SA-bNP to allow for visualization of particle and SA co-localization in the tissues. It has been reported that longer antigen residence times correlate with enhanced immune responses (33). To assess the extent to which bNP increased SA residence time, lungs and dLN were extracted from the mice and imaged at various timepoints, up to 14 days after administration. Image analysis shows that the fluorescence of SA alone decreases over time, whereas SA-bNP is highly fluorescent in the lungs and dLN and is detectable for up to 14 days (**Figure 3C**). SA alone, the SA in SA-bNP and the bNP in SA-bNP were quantified at days 1, 2, 3, 7, 10 and 14 post IN administration. The difference in fluorescence between SA alone and SA in SA-bNP in the lungs was significant at days 2, 5, and 7. Alternatively, no significant differences were observed in the measured values of bNP in SA-bNP at any timepoint (**Figure 3D**). Together, this data suggests that bNP improves the residence time of antigen in the lungs. Similarly, fluorescence of SA in SA-bNP vs SA alone in the lymph nodes trended higher across all time points, though they did not reach statistical significance (**Figure 3E**). The variability observed in the lymph nodes may be due to differential lymphatic drainage pathways of particles in the mouse model. Together, these patterns of elimination and accumulation indicate that bNP remains colocalized to loaded antigen and is retained in target tissues.

### 3.4 PreF-bNP is internalized by lung dendritic cells and macrophages after IN administration

**Figure 4:**
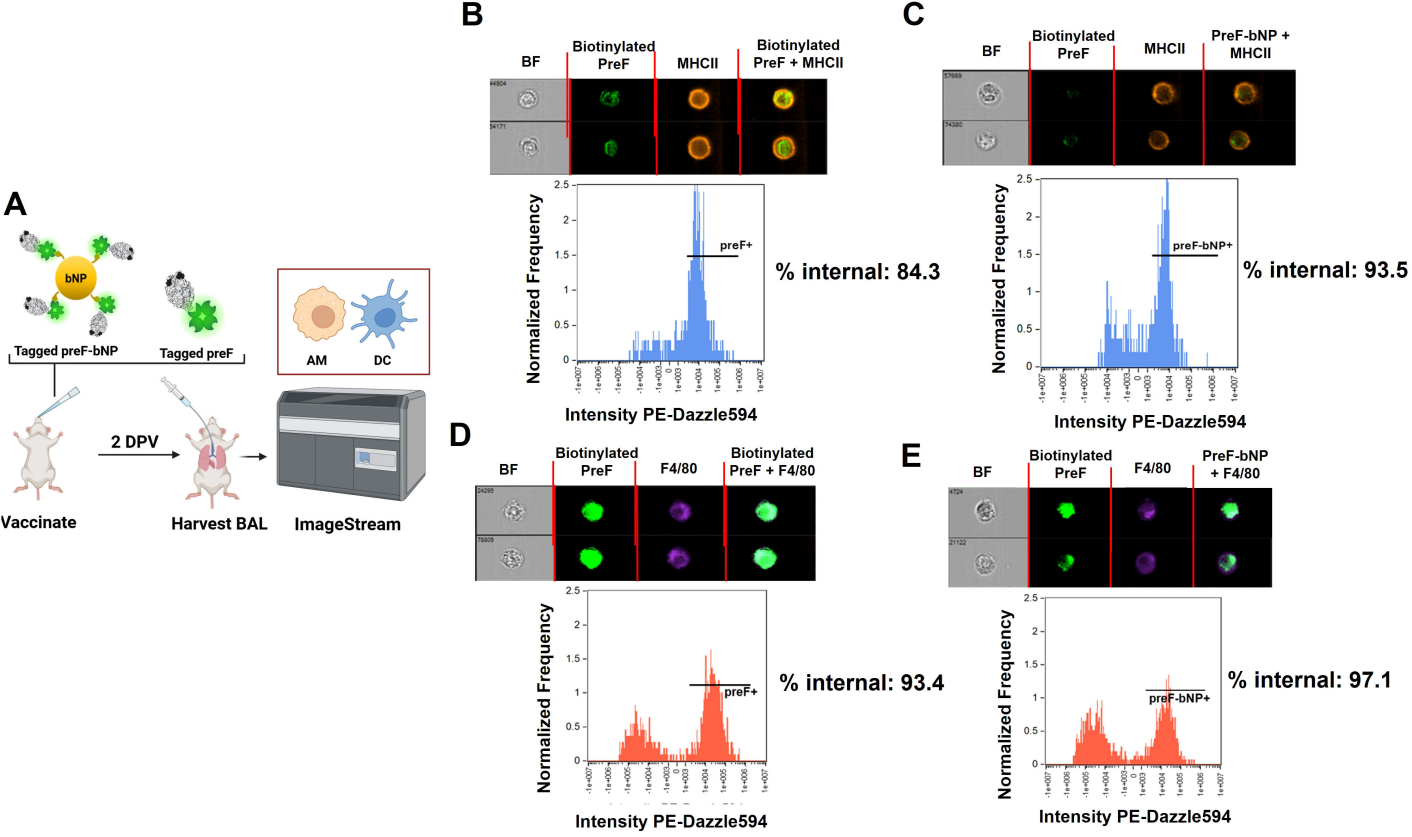
PreF-bNP is internalized by lung macrophages and DCs after IN administration. **(A)** Experimental workflow for ImageStream - created by using BioRender. **(B)** Representative DC images with fluorescence for biotinylated PreF, MHCII, and overlays with histogram of internalization of biotinylated PreF. **(C)** Representative images of macrophages with fluorescence of biotinylated PreF, F4/80, and overlay with histogram of biotinylated PreF internalization. **(D)** Representative images of DCs with fluorescence of PreF-bNP, MHCII, and overlay with histogram of PreF-bNP internalization **(E)** Representative images of macrophages with fluorescence of PreF-bNP, F4/80, and overlay with histogram of PreF-bNP internalization by DCs. %internal is calculated from Equation 1. BF= Brightfield. DCs were characterized by single cell/F480-/CD11c+/MHCII hi and Macrophages were characterized as single cell/F480+ via IDEAS software v6.3. N = 4 per treatment group. Two BAL from same treatment group were combined to create one sample. Only one representative sample is shown in the image.

Given the extended retention of SA-bNP in the lungs and dLN, we next sought to analyze the particle interactions with myeloid cells in the airways. Mice were intranasally administered biotinylated PreF or PreF-bNP suspended in PBS. Both formulations were fluorescently tagged with SA conjugated to PE-Dazzle594 for detection. Mice were preliminarily infected with RSV and 1 week later were intranasally vaccinated. Two days post vaccination, BAL from the separate cohorts of mice were stained for DCs and macrophages (**Figure 4A**). Internalization of PreF-bNP or PreF alone was measured via ImageStream^X^ MKII imaging flow cytometer and calculated using Equation 1 in the methods section. Within the representative data, 84.3% of DCs internalized PreF alone whereas 93.5% of DCs internalized PreF-bNP (**Figures 4B, C**). Alternatively, 93.4% and 97.1% of alveolar macrophages (AMs) internalized PreF alone and PreF-bNP, respectively (**Figures 4D, E**). These results suggest that bNP improves PreF internalization into DCs while retaining macrophage uptake efficiency.

### 3.5 PreF-bNP activation of the innate immune response

**Figure 5:**
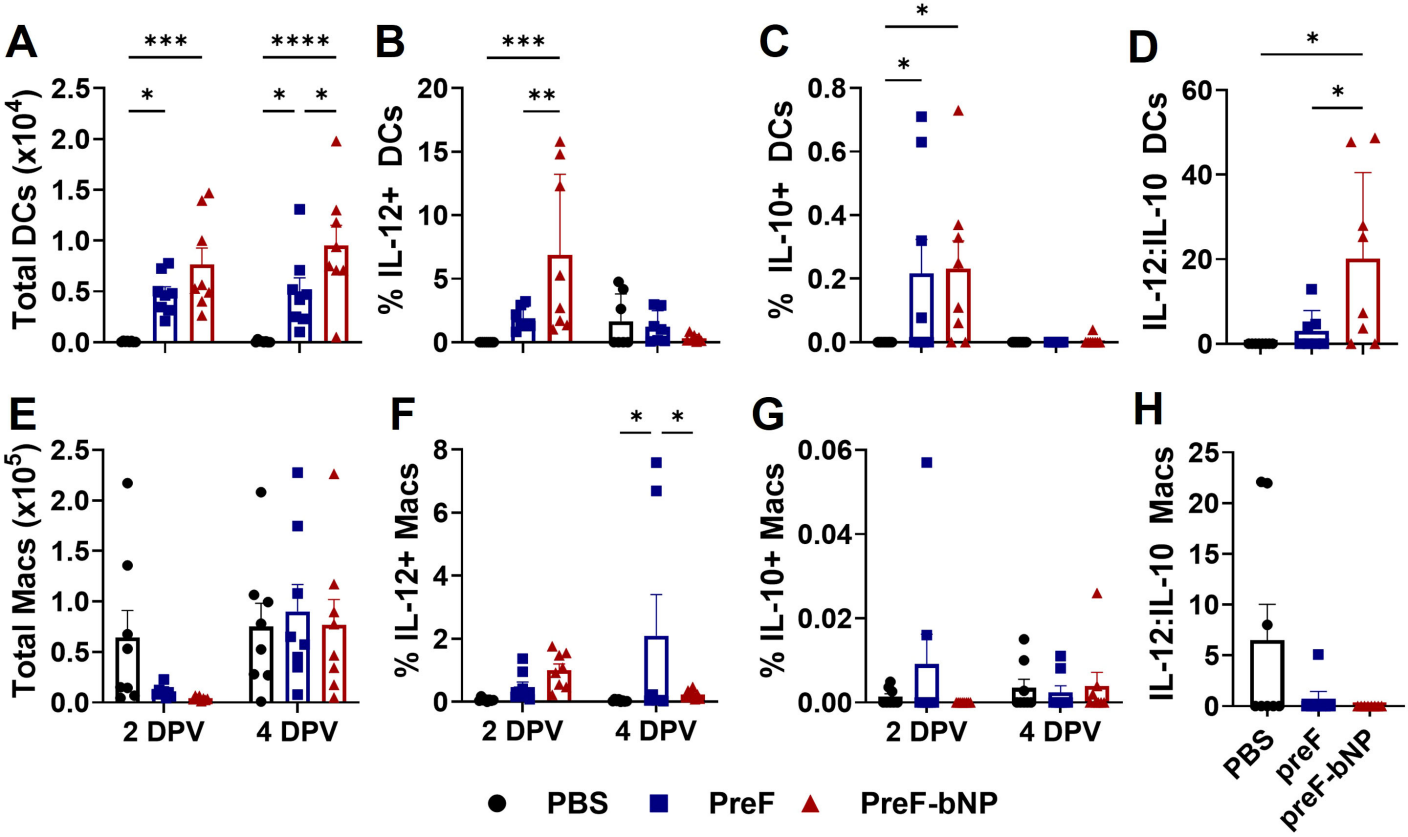
PreF-bNP increases DC response and skews towards a Th1 type response. **(A)** Total number of DCs for each group at 2- and 4-DPV. **(B)** Percent IL-12+ DCs. **(C)** Percent IL-10+ DCs. **(D)** Ratio of total IL-12+ DCs to total IL-10+ DCs at 2 DPV **(E)** Total number of macrophages at 2- and 4-DPV **(F)** Percent IL-12+ macrophages **(G)**. Percent IL-10+ macrophages **(H)** Ratio of total IL-12+ macrophages to total IL-10+ macrophages. N = 8 mice per group. * p<0.05, **p<0.01, ***p<0.001, ****p<0.0001 by two-way ANOVA with Tukey’s multiple comparison *post-hoc* test.

Next, we aimed to assess the extent of particle-mediated DC and macrophage activation. PBS, PreF alone (untagged, unbiotinylated), or PE-Dazzle594-tagged SA conjugated to PreF-bNP was delivered IN to preimmune 4-week-old BALB/c mice. BAL was collected at 2- or 4-days post vaccination (DPV) to quantify cell number and cytokine response of lung DCs and macrophages via flow cytometry (APC gating in **Supplementary Figure 3**). The number of DCs increased in the PreF-bNP group at both 2 and 4 DPV compared to PBS and PreF alone (**Figure 5A**). PreF-bNP also increased the functional antiviral activity of DCs as evidenced by a higher frequency of IL-12 to IL-10 expressing DCs at 2DPV (**Figures 5B–D**); little cytokine production was observed in either group at 4DPV. Total macrophages numbers were similar between groups (**Figure 5E**). A small increase in the percentage IL-12+ macrophages in the PreF alone compared to the PreF-bNP group was observed at 4 DPV with no significant change in IL-10 production (**Figures 5F, G**) and no difference in the frequency of IL-12 to IL-10 expressing macrophages (**Figure 5H**). Together, these data demonstrate that PreF-bNP improves uptake and activation by DCs, with minimal impact on alveolar macrophages.

### 3.6 PreF-bNP induces Th1 CD4 and antiviral CD8 T cells and confers protection against RSV

To determine if improved residence time and DC activation observed with PreF-bNP correlates with improved T cell responses and protection against RSV, preimmune young mice were vaccinated in a prime-boost protocol and challenged with RSV. Briefly, naïve BALB/c mice were bred for timed delivery of pups. At postnatal day (PND) 7, pups were infected with 2.5x10^5^ pfu/g of RSV-L19 to recapitulate early life RSV infections observed in children under 1 year of age and to elicit a viral-mediated Th2-type immune imprint – as reported by our lab and others (34, 35). Three weeks later, mice were vaccinated IN with 100μL of PBS, PreF alone, or PreF-bNP and boosted again 3 weeks later. Four or 8-weeks post-booster vaccination, mice were challenged with 4x10^5^ pfu/g of RSV-L19.

**Figure 6:**
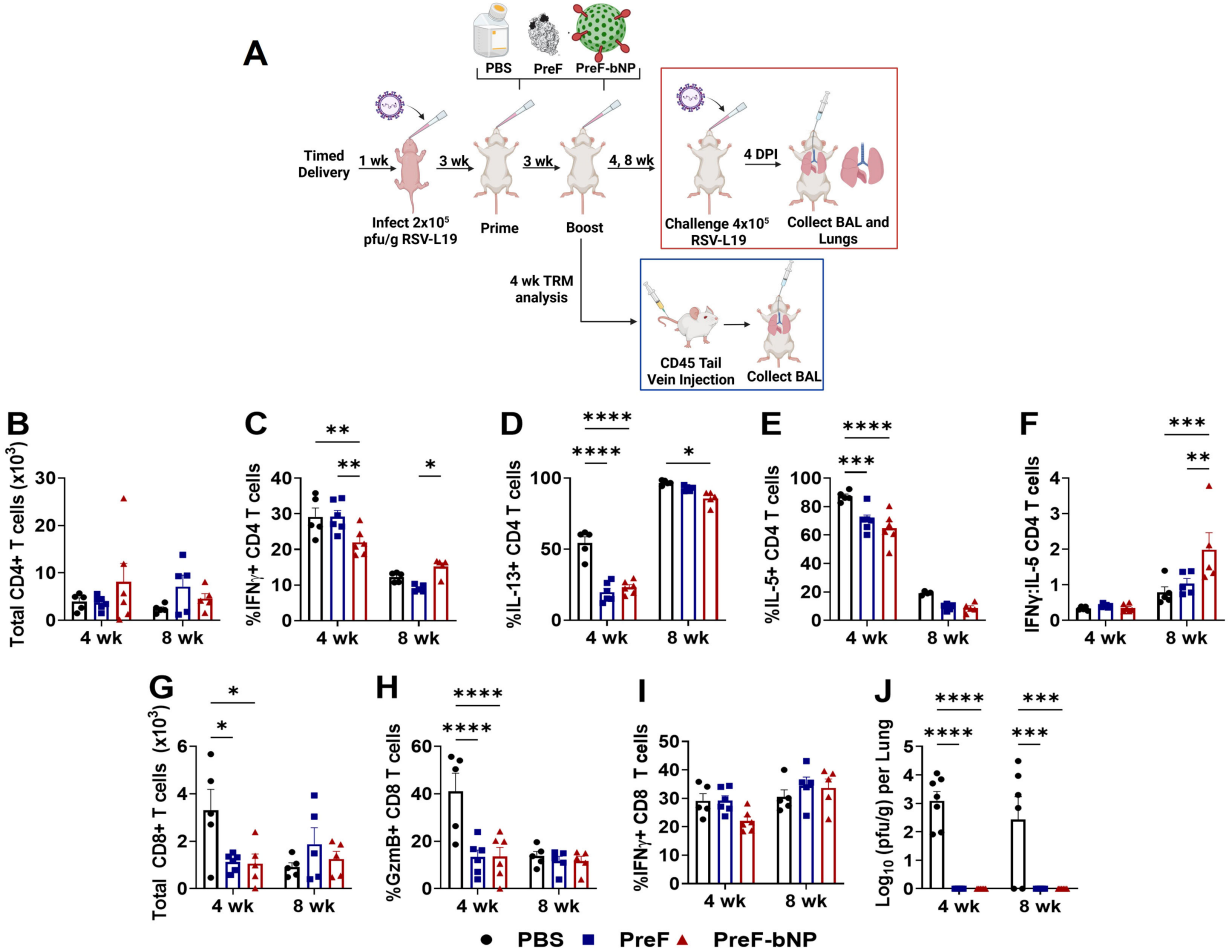
CD4 and CD8 T cell responses indicate a shift towards a Th1 type antiviral response and confer full protection against RSV 8 weeks post-boost with challenge. **(A)** Schematic of experimental design for vaccination schedule and T cell analysis - created by using BioRender. **(B)** Total number of CD4+ T cells at 4- and 8-weeks post boost and 4DPI. **(C)** %IFNγ+ **(D)** %IL-13+ and **(E)** %IL-5+ CD4 T cells. **(F)** The ratio of total IFNγ+ to total IL-5+ CD4 T cells. **(G)** Total number of CD8+ T cells **(H)** %Granzyme B+ and **(I)** % IFNγ CD8+ T cells at 4 and 8 weeks. **(J)** Viral titers measured in Log10 pfu/g per lung at 4 and 8 weeks post vaccination. N = 6 mice per group per timepoint. *p<0.05, **p<0.01, ***p<0.001, ****p<0.001 by two-way ANOVA with Tukey’s *post hoc* test for multiple comparisons.

At 4 days post infection (DPI) effector T cell responses were measured from BAL via flow cytometry (**Figure 6A**). Total CD4+ T cells were comparable among vaccinated and unvaccinated control mice at both 4 and 8 weeks (**Figure 6B**). However, differential cytokine profiles were observed. The %IFNγ+ CD4 T cells were lower at 4 weeks, but higher at 8 weeks in PreF-bNP- compared to PreF-vaccinated mice, suggesting that PreF-bNP elicits a measured, but durable Th1-type response (**Figure 6C**). Compared to PBS controls, %IL-13+ CD4 T cells were reduced in both PreF-bNP and PreF vaccinated mice at 4 weeks. While %IL-13+ CD4 T cells were relatively higher at 8 weeks amongst all groups, it was significantly lower in PreF-bNP vaccinated mice (but not PreF alone) compared to PBS controls (**Figure 6D**). Similarly, a significant decrease in %IL-5+ CD4 T cells was observed in both PreF and PreF-bNP treated animals compared to PBS controls (**Figure 6E**). Overall, the ratio of total IFNγ:IL-5 positive CD4 T cells was significantly higher in PreF-bNP compared to PreF alone vaccinated mice at 8 weeks (**Figure 6F**), suggesting that PreF-bNP (but not PreF alone) elicits a persistent Th1 phenotype immune response up to 8 weeks.

Interestingly, total CD8+ T cells were reduced in both vaccinated groups compared to PBS controls at 4 weeks and were comparable among all groups by 8 weeks (**Figure 6G**). The frequency of GzmB+ CD8 T cells followed a similar pattern, whereby vaccinated groups were reduced compared to PBS controls at 4 weeks and all groups were comparable by 8 weeks (**Figure 6H**). Alternatively, the frequency of IFNγ+ CD8 T cells was similar in vaccinated and control mice at 4 and 8 weeks (**Figure 6I**). Together, these data suggest that IN vaccination with PreF-bNP elicit a persistent Th1-skewed CD4 T cell response compared to PreF alone, whereas IN vaccination with both PreF and PreF-bNP reduced CD8 T cell recruitment to the airways.

Despite reduced antiviral CD8 T cell responses, all PreF and PreF-bNP vaccinated mice were fully protected against RSV replication with no detectable plaques observed up to 8 weeks after vaccination. (**Figure 6J**). Moreover, the increase in GzmB+ CD8 T cells observed in PBS mice failed to protect against RSV infection. These data suggest that circulating CD8 T cells found in the BAL may not significantly contribute to protection against acute RSV infection following IN vaccination.

### 3.7 PreF-bNP induces Th1 CD4 and antiviral tissue resident memory T cells in the lungs

**Figure 7:**
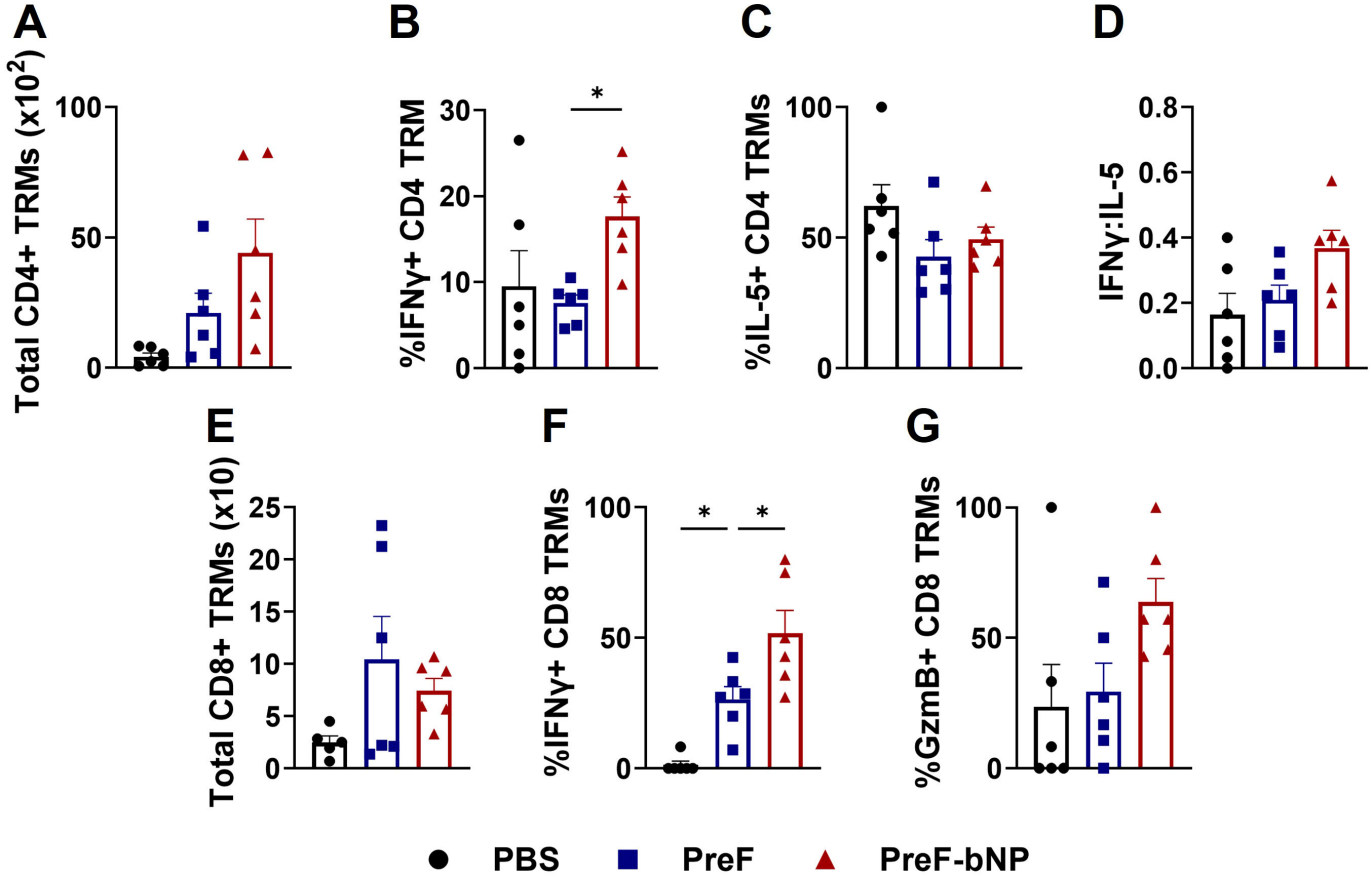
Tissue resident memory T cells (TRM) elevated 4 weeks post boost and show a skew towards Th1 type response. **(A)** Total CD4+ TRMs with **(B)** %IFNγ+ and **(C)** % IL-5+. **(D)** The ratio of total IFNγ+ and total IL-5+ CD4+ TRMs. **(E)** Total CD8+ TRMs with **(F)** %IFNγ+ and **(G)** %Granzyme **(B)** N = 6 mice per group per timepoint. * p<0.05 by one-way ANOVA with Tukey’s *post hoc* test for multiple comparisons.

To determine if IN vaccination with PreF-bNP promoted establishment of antiviral resident memory T cells (TRMs), a similar experimental approach was performed as shown in **Figure 6A**, except that lungs were harvested from RSV unchallenged mice 4 weeks after vaccination. To discriminate TRMs against circulating T cells, anti-CD45 antibody was injected via tail vein 3 minutes prior to harvesting lungs for flow cytometry (TRM gating strategy shown in **Supplementary Figure 4**). Total CD4+ TRMs trended higher for PreF-bNP treated animals compared to PreF and PBS control (**Figure 7A**). The frequency of IFNγ+, but not IL-5+ CD4 TRMs, was also increased in mice vaccinated with PreF-bNP (but not PreF) compared to PBS controls (**Figures 7B, C**), resulting in an increased trend of the IFNγ to IL-5 ratio among PreF-bNP compared to PBS and PreF vaccinated mice (**Figure 7D**). Unlike CD4 TRMs, total CD8 TRMs were similar across all groups (**Figure 7E**). However, the frequency of IFNγ+ CD8 TRMs was increased in mice vaccinated with PreF-bNP compared to PreF and PBS vaccinated mice (**Figure 7F**). A similar increasing trend in %GzmB+ CD8 TRMs was observed in PreF-bNP mice (**Figure 7G**). These data suggest that while the total number of CD8 TRMs were not significantly increased, the frequency of functionally active antiviral CD8 TRMs were increased by vaccination with PreF-bNP as compared to PreF or PBS.

### 3.8 Intranasal PreF-bNP mitigates mucus overproduction in the lungs

**Figure 8:**
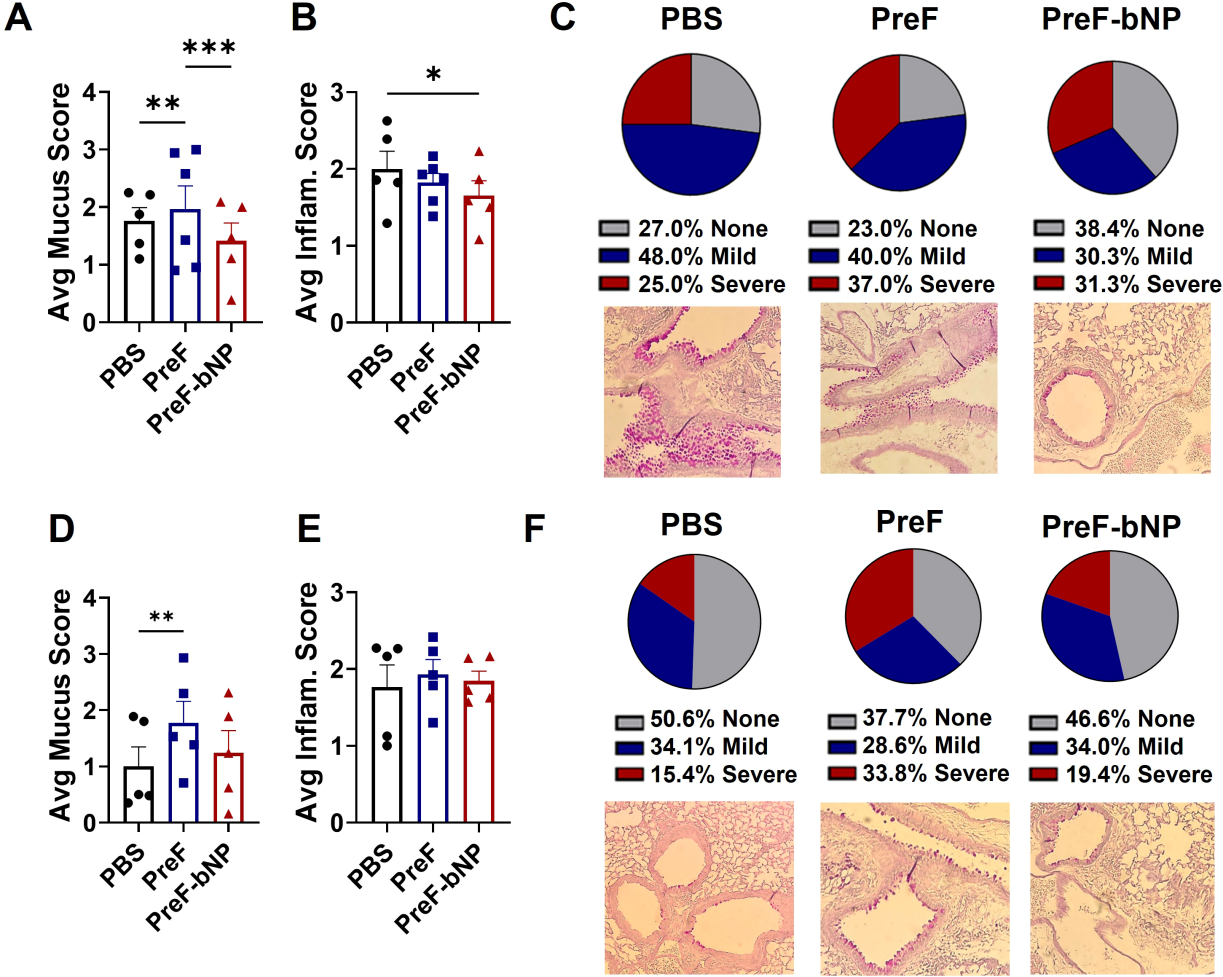
PreF-bNP has improved safety profile compared to PreF alone up to 8 weeks post vaccination. **(A)** Average mucus scores at 4 weeks **(B)** Average inflammation scores at 4 weeks **(C)** Distribution of severity of mucus overproduction in airways of PBS, PreF, and PreF-bNP lungs and representative PAS stain slide **(D)** Average mucus scores at 8 weeks. **(E)** Average inflammation scores at 8 weeks. **(F)** Distribution of severity of mucus production of PBS, PreF, and PreF-bNP lungs and representative PAS stain slide. Samples were analyzed via one-way ANOVA with Tukey’s *post hoc* test for multiple comparisons. N = 6 mice per group per timepoint. *p<0.05, **p<0.01, ***p<0.001.

To assess safety among the vaccination groups, lungs were harvested 4- and 8-weeks after vaccination following RSV challenge (as described in **Figure 6A**). Lungs were formalin filled and stained by Periodic Acid-Schiff (PAS) stain or hematoxylin and eosin (H&E) at 4dpi to assess mucus production and inflammation, respectively. At 4 weeks, average mucus and inflammation scores were reduced in mice vaccinated with PreF-bNP compared to PreF or PBS vaccinated mice (**Figures 8A, B**). Moreover, severity of mucus production (severe = 50-100% of mucus coverage in an airway), was also lower in PreF-bNP mice versus PreF mice (**Figure 8C**). Average mucus scores in PreF vaccinated mice (but not PreF-bNP vaccinated mice) were increased through 8 weeks post vaccination, whereas PreF-bNP was similar to PBS controls (**Figure 8D**); inflammation scores were similar across groups (**Figure 8E**). Importantly, the mucus severity score was dramatically lower in PreF-bNP (19.4% compared to PreF alone at 33.8%) as shown in the pie charts and representative images of PAS staining (**Figure 8F**). Overall, these data indicate that mice vaccinated with PreF-bNP have an enhanced safety profile with reduced inflammation and mucus overproduction compared to PreF alone.

## 4 Discussion

We report here evidence that IN administration of an RSV subunit vaccine formulated with bNP is effective in inducing an antiviral and Th1 skewed viral-specific immune response in juvenile preimmune mice. The PLGA-based bNP formulation described here is unique because the design consists of an affinity capture mechanism (biotin-streptavidin) for loading antigens on the particle surface, rather than through encapsulation (21). Surface loading has the advantage of circumventing protein denaturation seen in common encapsulation processes that involve organic solvent and shear stress (36). Consistent with these previous findings, our data show that PreF retained its conformation on the surface of bNP for up to 4 weeks in PBS. The observed conformational stability is consistent with the computational modeling which shows the predicted distance between each biotin binding site is larger than the size of PreF, suggesting it is unlikely for PreF molecules to overlap or sterically interfere with each other. This allows for antigen to remain in its native conformation while being recognized by B cells and be taken up by DCs.

The stable complexation of PreF on the bNP surface is a critical attribute of the particle formulation *in vivo* because free antigens are rapidly cleared shortly after injection (<24h) (36), thus limiting the induction of downstream immunity. The duration of the PreF-bNP vaccine residence within the tissue allows for sufficient time for interaction with and activation of DCs leading to the production of resident memory T cells (37, 38). When streptavidin [used as a mock antigen (16)] was IN administered by itself, it was rapidly cleared from the lungs. When formulated with bNP however, SA-bNP was retained within the lungs and draining lymph nodes for up to 14 days after intranasal administration, suggesting bNP enhances antigen residence time in the lung. Moreover, PreF-bNP increased IL-12+ DCs and production of Th1-cytokine producing CD4 and antiviral CD8 TRMs as compared to animals vaccinated with PreF alone. Being that prolonged antigen residence time promotes establishment of TRMs (33, 39) the increased TRM response observed indicates that bNP increases the residence of time of PreF allowing for the enhanced DC activation and generation of TRMs.

The analysis of initial DC uptake of the vaccine and subsequent activation *in vivo* supports the notion that intranasal administered particle-based vaccines may enhance DC uptake compared to less efficient antigen presenting cells (i.e. macrophages). Internalization of the vaccine particles was confirmed using ImageStream (40), which indicated DCs internalized a higher fraction PreF-bNP compared to PreF, thus confirming that bNP promotes APC uptake in the lung following IN delivery. Consistent with the increase in IL-12+ DCs, Th1 CD4 and antiviral CD8 effector T cell responses were also increased in mice treated with PreF-bNP compared to PreF alone, indicating that Th1-skewed immunity was consistent across innate and adaptive immune responses.

In young children, RSV infection promotes a predominant Th2 response leading to enhanced mucus production and inflammation (41, 42). Vaccines adjuvanted with Th2-type adjuvants, such as alum, may also exacerbate the type 2 inflammatory response (43) and potentially worsen disease outcomes. We previously reported that intramuscular delivery of the RSV prefusion F protein alone also enhanced Th2-mediated mucus over-production and inflammation following RSV challenge (44). It is thus critical for an RSV vaccine to promote a strong antiviral Th1-polarizing response to avoid disease exacerbation. Delivery of vaccines intranasally have the added requirement of overcoming immune tolerance in the nasal mucosa, further justifying the need for a strong Th1-polarizing vaccine. We previously reported that the bNP platform possesses inherent Th1-polarizing properties (15, 16), which are translated in the current studies where PreF-bNP elicited a Th1 dominant response with reduced average mucus and inflammation scores compared to PreF alone. The addition of Th1-skewing adjuvants will likely extend viral protection and reduce mucus production.

This is the first reported study to examine intranasal delivery of a nanoparticle RSV vaccine in a preimmune neonatal mouse model (45, 46). Immunization of mice previously exposed to RSV mimics the proposed target population for this vaccine strategy: children ~3 years of age who were previously exposed to RSV. Direct vaccination of RSV naïve children increases the risk of vaccine enhanced disease, and due to their young age, it is unlikely to elicit long-term protection (47). We previously showed that IM administered PreF plus a type 1 adjuvant could overcome the RSV-mediated Th2-skewed immune imprint in young mice (48). Results reported here show that IN delivery of PreF-bNP in preimmune mice overcome the Th2 imprint associated with early life RSV infection and promote a Th1-phenotype in DCs (IL-12), increase the IFNγ to IL-5 ratio in CD4 helper T cells and increase Th1-mediated cytotoxic CD8 T cells in preimmune mice. Together, these data support the use of bNP as an IN-RSV subunit vaccine platform for inducing an antiviral and Th1 skewed immune response against RSV.

## Data Availability

The raw data supporting the conclusions of this article will be made available by the authors, without undue reservation.
