# Supplementary figures and images for: Intranasal delivery of a polymeric nanoparticle subunit vaccine for the induction of protective immunity against respiratory syncytial virus
Source: Front Immunol. 2026 Jun 26;17:1824823. doi: 10.3389/fimmu.2026.1824823 (PMC13350243; doi:10.3389/fimmu.2026.1824823)

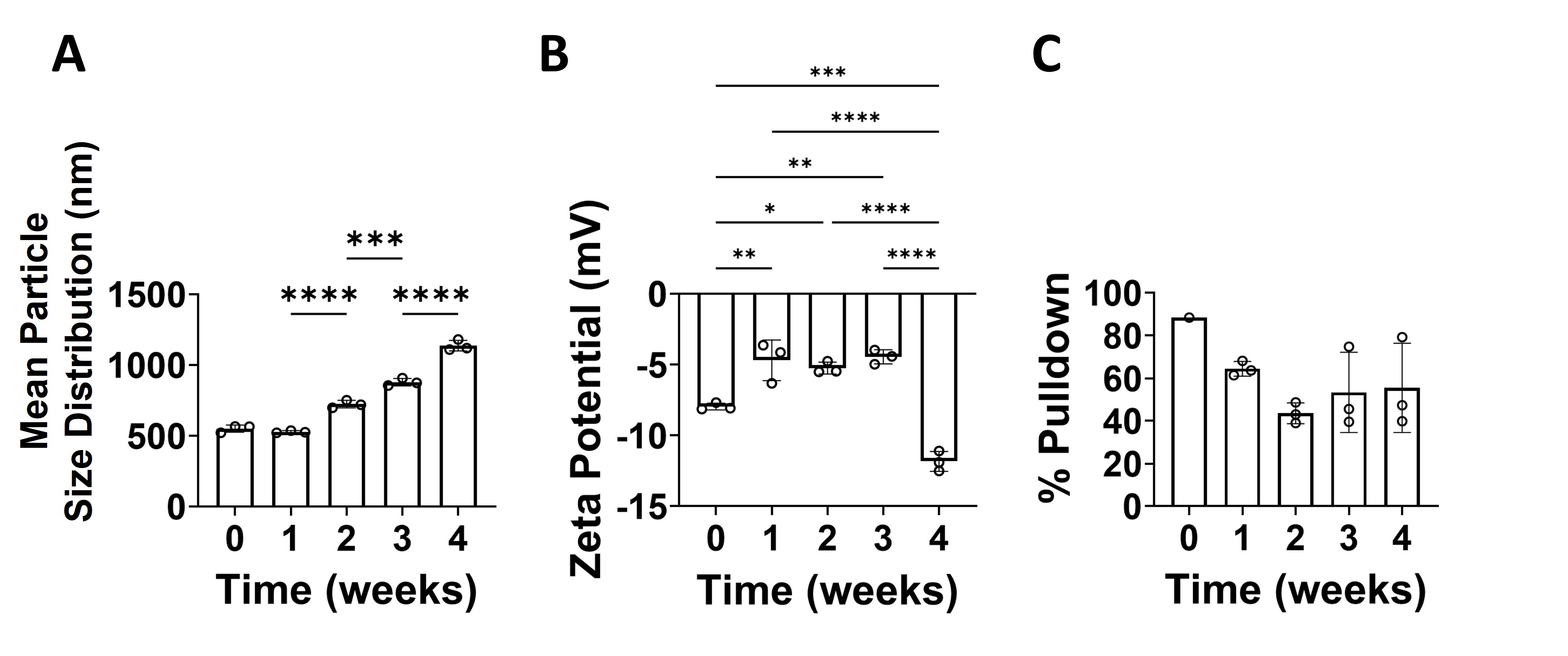

Supplement: Supplementary file 1 [file Image1.jpeg]

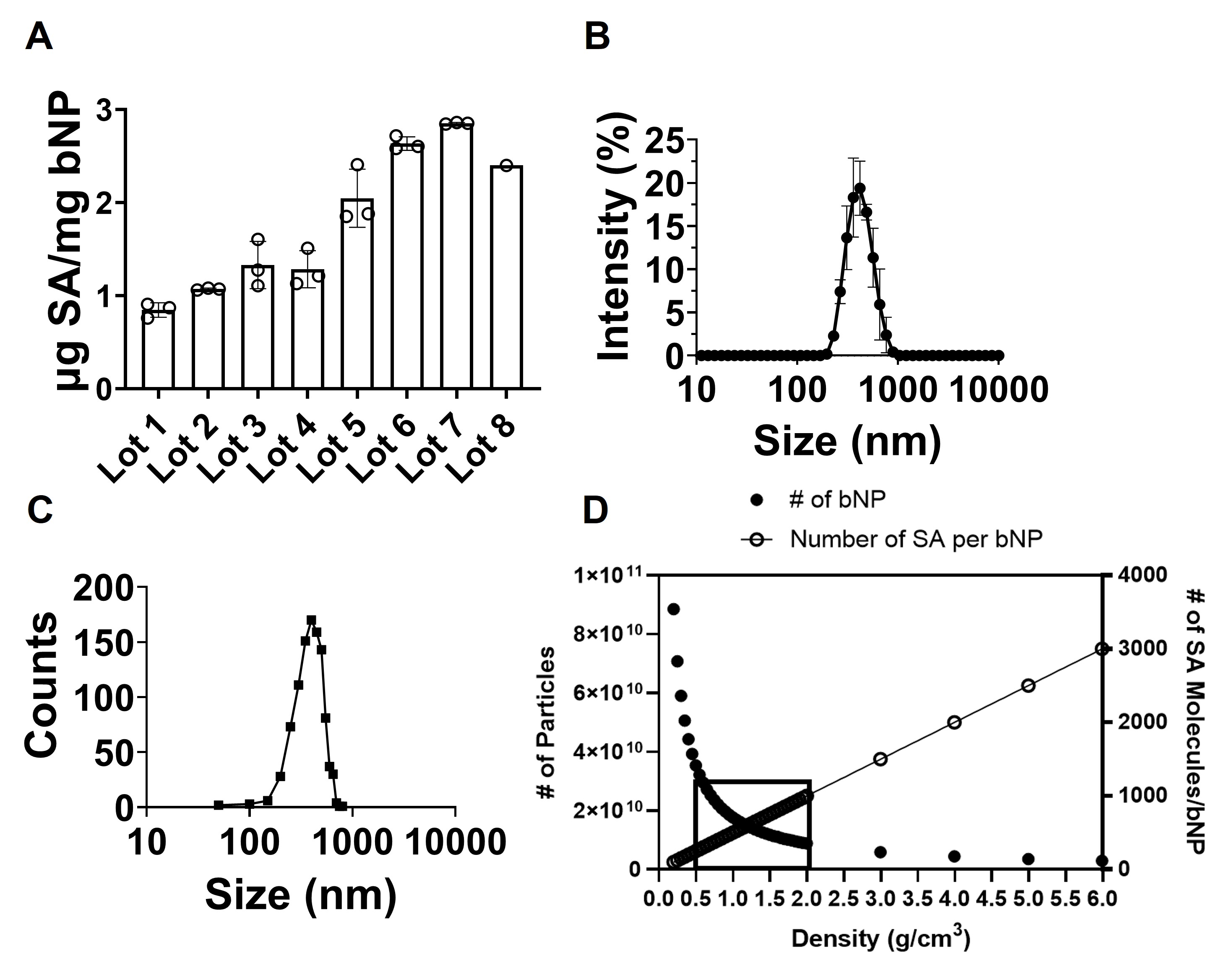

Supplement: Supplementary file 2 [file Image2.jpeg]

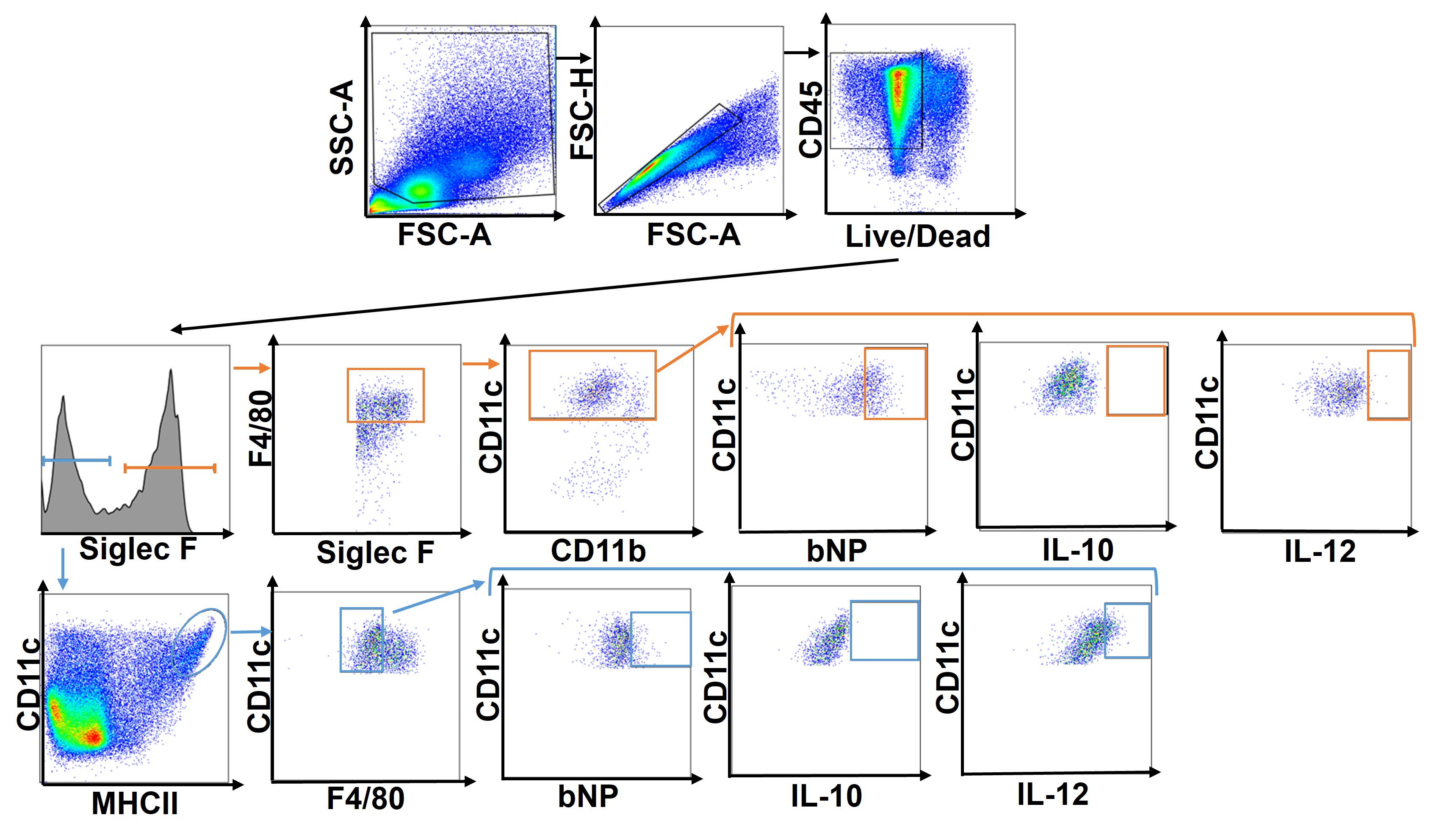

Supplement: Supplementary file 3 [file Image3.jpeg]

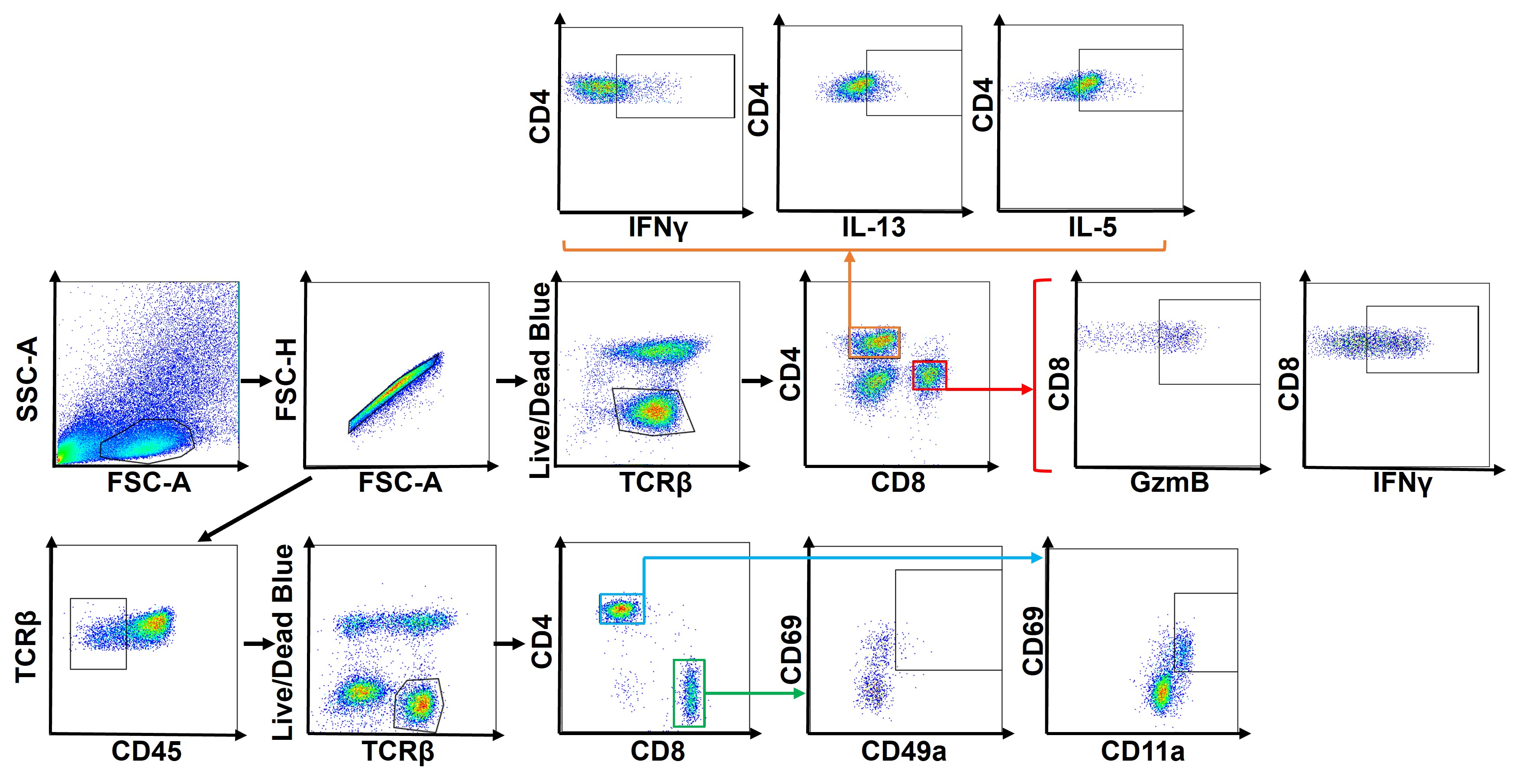

Supplement: Supplementary file 4 [file Image4.jpeg]
